# Sex determination and *Aedes* population control

**DOI:** 10.1186/s13071-018-3217-6

**Published:** 2018-12-24

**Authors:** Helena Rocha Corrêa de Araújo, Bianca Burini Kojin, Margareth Lara Capurro

**Affiliations:** 1 Department of Parasitology, Institute of Biomedical Sciences, University of São Paulo, São Paulo, Brasil; 2 Department of Entomology, Texas A & M University, Minnie Belle Heep Center, College Station, TX USA

**Keywords:** *Aedes aegypti*, vector borne diseases, mass-rearing and population suppression

## Abstract

The global economic cost of *Aedes*-borne diseases, such as dengue, is estimated to be in the billions of dollars annually. In this scenario, a sustained vector control strategy is the only alternative to control dengue, as well as other diseases transmitted by *Aedes,* including Zika and chikungunya. The use of transgenic mosquitoes is a promising weapon in the improvement of approaches currently applied in *Aedes aegypti* control. Field trials using genetically modified mosquitoes for population control have been conducted and offer an excellent opportunity to evaluate what can be improved. In a mass-rearing mosquito facility, the absence of a transgenic line that produces male-only progeny is undoubtedly a limiting factor; thus, being able to manipulate sex determination in this species is a fundamental step for the success of this strategy. Likewise, the possibility of manipulation of the sex determination pathway opens-up a new opportunity for disease control.

## Background

The World Health Organization estimates that approximately 50–100 million dengue infections occur annually in more than 100 countries. Over the past 50 years, dengue has been the mosquito-borne disease that has most rapidly spread around the world, increasing global incidence by 30-fold [1]. The global economic cost of dengue is measured in the billions of dollars every year in the Americas and South-East Asia [2, 3]. The effect of dengue control costs was evaluated in six countries from America and Asia (Brazil, Columbia, Malaysia, Mexico, the Philippines, and Thailand) over a 15-year period in a hypothetical scenario that would use a medium-efficacy and low-cost immunization strategy. If a high-efficacy vector control was used in this hypothetical scenario, based on existing technologies, the vector population would decrease by 70–90%, and the cost averted per disability-adjusted life year (DALY) would be $ 679–1331 USD (best estimates), considering direct medical and control programme costs, relative to no intervention [4].

The recent spread of Zika virus (ZIKV) in 47 countries and territories in the Americas [5] has revealed a major concern to health authorities around the world because of the unusual health impacts not previously observed after arbovirus infections, such as congenital ZIKV infection syndrome [6] and Guillain-Barré syndrome [7]. In addition, five countries in the Americas (Argentina, Canada, Chile, Peru, and the United States of America) reported sex-acquired ZIKV cases [5, 8]. Chikungunya virus (CHIKV) has been reported in at least 42 countries in North and South America, including the United States [9]. The main mode of ZIKV and CHIKV transmission to humans is through the bite of infected *Aedes* mosquitoes. Since there are no effective vaccines or specific drugs to prevent or treat these diseases, the only line of defense is to limit contact between mosquitoes and humans through vector control.

Since 2016, Brazil has been experiencing a massive outbreak of yellow fever (YF) in several regions of the country, such as Minas Gerais, Espírito Santo, Rio de Janeiro, Bahia and São Paulo, including areas where YF was not considered a risk [10, 11]. There are several factors that could be contributing to the current YF outbreak, such as climatic conditions, urbanization, high population mobility across the country, and the recent economic crisis that has impacted infrastructure, vector control and other public health programmes [12]. This situation highlights that sustained vector control is still the best alternative to fight multiple diseases transmitted by the same mosquito species.

## Integrated vector management (IVM)

The occurrence of frequent outbreaks in recent years caused by viruses transmitted by *Ae. aegypti* serves to highlight those conventional control methods that involving chemical, mechanical and biological control are not enough to combat vector-borne diseases, necessitating the application of novel vector control technologies [13].

IVM promotes the use of a range of interventions, based on knowledge about the vectors and diseases, to optimize the use of resources and tools for vector control [14]. Several countries around the world are showing increased interest in the use of the sterile insect technique (SIT) as an integrated approach in the management of vectors that transmit diseases, such as mosquitoes. SIT is a type of biological pest control that uses ionizing radiation (gamma rays or X-rays) to promote sterilization of the male insect. The mating of released sterile males with native wild females may lead to a decrease in reproductive potential and contribute to local suppression of the vector population if the number of males released is sufficient and occurs during the necessary time [15, 16]. Sterile males should be released weekly to maintain a permanent population in the target area so that females have a high chance of mating with a sterile male [17]. The frequency and number of sterile males released has to be carefully assessed in relation to the average longevity of the sterile males [18]. SIT has been effective for the containment, suppression or eradication of several major insect pest species in various parts of the world with numerous successful cases [19]. This technique would be combined with others, as part of the IVM approach to reduce the mosquito population.

Another example includes the exploration of the natural phenomenon known as cytoplasmic incompatibility (CI). Many diplo- diploid species express an embryonic lethality after mating occurs between the intracellular bacterial symbiont *Wolbachia*-infected males and uninfected females or females infected with a different *Wolbachia* strain [20, 21]*.* CI-based population suppression is known as the incompatible insect technique (IIT), and this technique can be used alone or in combination with SIT (IIT/SIT) to suppress mosquito populations [22]. In 2014, a pilot field trial was made in Lexington, Kentucky, USA using *Wolbachia* bacteria-infected males of *Aedes albopictus*. The local population of this mosquito species showed a localized reduction based on analyses of egg hatch and adult female numbers [23].

In IIT, the separation of males and females prior to release is particularly important, because the accidental release of infected females may result in replacement of the targeted population, instead of the intended suppression. Thus, a strategy combining SIT with IIT is the best alternative. In IIT/SIT, complete sterility in males would be ensured by both irradiation and *Wolbachia* infection, while a low irradiation dose is required to produce complete sterility in females and prevent population replacement [15, 22, 24, 25]. In China, a combined IIT/SIT was used for *Ae. albopictus* under semi-field conditions and the results are encouraging for further use of this strategy [26].

Other technologies that focus on mosquito population suppression were developed and tested in pilot trials. The Release of Insects carrying a Dominant Lethal (RIDL) was evaluated in the laboratory and in open-field trials in two municipalities of Brazil between 2010 and 2015. The *Ae. aegypti* transgenic male mosquitoes known as OX513A, that carry a gene that induces offspring death during the larval stage, were used in an attempt to suppress the mosquito population. In both trials, there was an average suppression of 70% in *Ae. aegypti* local populations compared to the prerelease period [27–29].

Mathematical models have demonstrated that strategically combining the suppression methods of SIT and RIDL with *Wolbachia* can generate a sustained control while mitigating the risks of inadvertent exacerbation of the wild mosquito population [30]*.*

To reduce mosquito populations or to replace competent vector populations (with a disease-refractory population), the use of genetically modified mosquitoes must guarantee the release of males for implementation to be efficient. To date, there has not been an *Aedes* mass-rearing facility which can provide males for release without the risk of accidental female contamination. To this end, an efficient method of sex separation in mosquitoes is necessary for the success of vector control methods that rely on the mass release of male mosquitoes.

## Why alter the sex ratio of *Ae. aegypti?*

It is important to manipulate the sex determination pathway in *Ae. aegypti* because for control purposes, it is mandatory to release only male mosquitoes in a mosquito release programme. This is mainly because female mosquitoes are the blood-feeders and disease vectors, and the release of female mosquitoes would be a nuisance to the human population.

In an area-wide integrated pest management (AW-IPM) that uses the SIT to release sterile males of the fruit fly, *Ceratitis capitata*, the application of genetically sexing strains (GSS) increases the effectiveness of the programme. The release of sterile females does not contribute to the sterility of the target population, and the production of females by the medfly mass-rearing facility is inopportune. In addition, the main drawback to releasing sterile medfly females is the damaging activity by these females of injecting embryos into the fruit, which can occur even if they are sterile. The same situation is present with disease vectors, as females are not required to transfer sterility to the target population; moreover, females are not releasable due to their capacity to transmit diseases even when they are sterile [31–33].

In a bisexual mosquito strain mass-rearing facility, there are several steps for male production and release. As mentioned before, the bisexual strain requires sex sorting, including segregation of the larvae from the pupae, and then finally separating the male from the female pupae.

This process is made possible through the use of mechanical sex separation, using a sorter known as a glass-plate separator [34]. This sex separation technique utilizes the distinct size between *Ae. aegypti* male and female pupae [35]. The sorting procedure starts when trays containing larvae and pupae are spilled between vertical glass plates that form a size gradient, and the pupae are carefully washed by adjusting the angle and distance between the plates. With the continuous water flow, the larvae are flushed, followed by the male pupae, and lastly the female pupae [36].

The RIDL strain of *Ae. aegypti*, OX513A, tested in Brazil was mass-reared in Biofabrica Moscamed Brasil, located in Juazeiro city, Bahia, Brazil. The Moscamed facility produced at most 1.5 million transgenic male mosquitoes per release. This number of mosquitoes was achieved with a dedicated egg colony producing approximately 4 million eggs weekly. In the mass-rearing facility proposed by [36], the egg production colony must be of a sufficient size to provide the number of eggs needed for the weekly release. It is recommended to provide eggs sufficient for four weeks’ release, as well as colony maintenance. In addition, the demand for male mosquitoes will determine the size of this colony [36]. In this mass-rearing facility, the large-scale separation was performed using the glass-plate separator method mentioned before, five days per week over the eight-hour workday. That meant that a unique step consumed most of the time required to rear the mosquitoes, which was also reflected in its costs.

The glass-plate method has another huge inconvenience. Approximately 0.5–1% of the mosquitoes are physically misidentified during the sex-sorting step, due to factors related to larval tray density, feeding regime, rearing temperature and the egg hatching procedure. Therefore, it is essential to check for female contamination after sorting. The Brazilian facility used three aliquots containing 500 pupae each, that were recently sex-sorted, and the number of females present was recorded. The dimorphism present in the anal segment of the pupa was used as the parameter to distinguish males from females in the contamination check [37]. The Brazilian project determined that no more than 1% of females present in the adult storage used for release was acceptable [36]. The quality control mentioned in the trial highlights the added cost of employee time and resources.

**Fig. 1:**
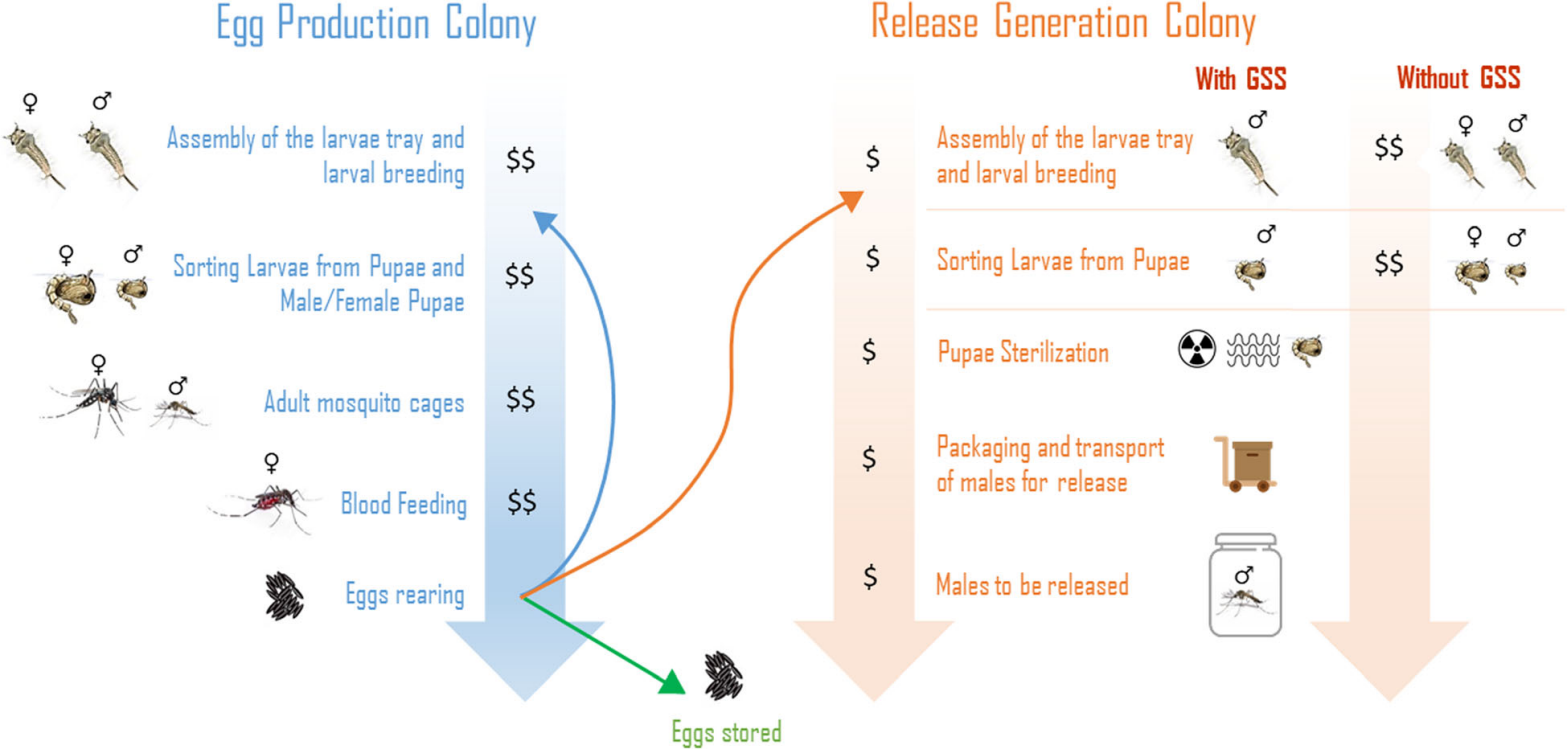
Stages to producing mosquitoes in a hypothetical mass-rearing facility with GSS. In a mosquito factory, mass rearing can be divided into two independent parts: rearing for colony egg production and rearing for male releases. The egg production colony produces equal numbers of the two mosquito sexes being necessary to perform the current method of sex-separation in mosquitoes using a glass-plate separator. The sorting of male/Female Pupae is performed because the colonies are formed in the ratio 1 male to 3 females. In a SIT mosquito release program that uses mosquito GSS, the step of larval production and sorting male/female pupae will not be necessary because the production of male-only progeny results in a low operating cost ($) compared to rearing males and females ($$)

We must further emphasize that approximately 50% of pupae produced in every batch for release were females that would have to be discarded, representing a significant waste in the production. Regrettably, these females remaining after sex-sorting cannot be put back into the egg production colony because the rearing of mosquitoes for the two colonies (egg production and release generation) are different, generating mosquitoes with different productive performances. However, an expense reduction is achievable using a genetic sex-sorting, such as a mosquito transgenic line that is able to produce male-only progeny. GSS can significantly contribute to a more specific and efficient process (Fig. 1), in addition it can be combined with other methods to optimize separation between the sexes [27, 28].

The Moscamed trial emphasized the need for a more precise method to alter the sex of transgenic mosquitoes to combat disease in control programmes with regards to cost, time, and safety efficiency.

One great example is an insect pest that parasitizes animals, the New World screwworm, *Cochliomyia hominivorax*. This pest was eradicated from all of North and Central America using SIT. The eradication programme released both sterile males and females, but since the start of the programmme 60 years ago, scientists have recognized the benefits of a male-only strain for population suppression [38, 39].

In 2016, a transgenic male-strain of *C. hominivorax* was developed by Concha and collaborators. This transgenic line is conditional female lethal and allows both sexes to be efficiently reared under permissive conditions, while only males are produced under restrictive conditions [40]. The use of this strain could lead to considerable savings in the production cost at the mass-rearing facility, once fewer insects are needed to maintain the eradication zone.

In addition to the substantial advantage that producing male only mosquito strains can bring to a mass-rearing facility for vector control using either RIDL or SIT, the sex distortion can also be used as an approach for the population suppression of pests. It is hypothesized that releasing males that would only produce male offspring would produce a population collapse. Two different approaches to sex ratio alteration were modeled. In the first, males were released carrying a dominant-male determined genetic element that would only produce genetically male offspring; in the second, the females’ offspring from released males are phenotypically transformed into males. Both approaches may be at least two orders of magnitude more efficient than sterile male releases (SIT) regarding the number of surviving insects [41]. These two approaches are still the subject of research, and a final product remains to be generated and tested for efficacy.

## What we know about sex determination in *Ae. aegypti* and strategies for sex alteration?

The whole molecular aspect of sex determination in *Ae. aegypti* is still an ongoing subject of research. To date, only a few genes have been characterized and known to act as determinants in this pathway.

The *Ae. aegypti* karyotype has three pairs of chromosomes, numbered 1, 2 and 3, and a heteromorphic sex chromosome is absent in this species. In the centromeric region of chromosome 1 lies the M locus, which is responsible for sex determination [42]. However, it was not long ago that the gene responsible for initiating the male development for this species (described as Nix) was discovered. Nix cDNA is 985 base pair long region coding a 288-amino acid polypeptide. It is present in male genomic DNA but not in the female, its expression correlates to the stage before sex is determined, and finally, Nix is located within the M locus [43].

Downstream from Nix, in the sex differentiation pathway, two other genes have been characterized: *Fruitless* (*fru*) and *Doublesex* (*dsx*). *dsx* is responsible for the appropriate sexual differentiation of somatic cells. In *Ae. aegypti*, it produces two transcript isoforms in the female and only one in the male. This regulation is based on exon skipping in both male and female isoforms, 5’ alternative splice site choice, and two potential alternative polyadenylation sites [44]. *fru* also produces sex-specific transcripts via a conserved splicing regulation based on two 5’ alternative splice sites [45] and its expression is essential to sexual behavior in *Drosophila* [46], although within mosquitoes, functional tests to confirm the role of the *fru* gene in courtship behavior are still missing. In the presence of Nix, the *dsx* and *fru* genes produce the male-specific transcript through alternative splicing, and in its absence produces the female transcript [43].

In *Drosophila melanogaster,* the alternative splicing that produces the female-specific isoform for *dsx* is activated by a protein complex that includes *tra* and *tra -2* [47]. In *Ae. aegypti,* the binding sites for these two proteins, in addition to being present, their sequence conservation was low and was not comparable to the sequence conservation of the homologous elements identified in other dipteran species, where the splicing regulation of *dsx* and *fru* is under the control of *tra* and *tra-2* proteins [44, 45]. Based on the nature of sex determination to rely on alternative splicing, it might be possible that there is a *tra*-like protein in *Aedes* mosquitoes, but they have not yet been determined. One study has shown that knockdown of the homolog of *tra-2* in *Ae. aegypti* causes segregation distortion, but a functional study of this protein needs to be done to clarify its role in the sex determination pathway [48].

Currently, research groups around the world are conducting several studies to overcome mosquito sex sorting before male release. One example is the use of small interfering RNA mediated *dsx* silencing to target different sequences in exon 2 during *Ae. aegypti* pupal development. This method disrupted multiple sex-specific traits. Morphological, physiological, and behavioral alterations were observed in adult females, including decreased wing size and proboscis. Female lifespan, fecundity, and fertility were also significantly reduced. In addition, disruption of the olfactory system development was also observed. In other words, *dsx* regulates sexually dimorphic neural development in *Ae. aegypti* and is involved in the developmental control of sex-specific somatic properties [49].

RNAi-mediated knockdown of the female-specific isoform was effective in producing a highly male-biased population of mosquitoes when *dsx* double-stranded RNAs were delivered to larvae via soaking or being fed. However, no significant increase in the number of males was observed, indicating that there was female lethality, but no sexual conversion [50].

The discovery of Nix also provides an opportunity to manipulate the sex determination in the mosquito *Ae. aegypti*. The injection of a plasmid expressing Nix under the control of the *Ae. aegypti* polyubiquitin promoter generated more than 60% masculinized females. Unfortunately, complete sex conversion was not achieved in this transient assay [43]. Engineering the components of a transgene containing Nix could be an alternative to generating a complete sex conversion.

Finally, an example of successful sex conversion was achieved in the medfly *C. capitata*, after the discovery of the autoregulation of the *tra* gene in this species [51]. In *C. capitata*, differently from *Drosophila*, the *tra-2* gene is also involved in the splicing regulation of the *tra* gene [52]. RNAi against *tra* and *tra-2* led to the complete sex reversal of females to fully fertile and viable males [51, 52]. A *C. capitata* transgenic strain that is able to produce male-only progeny is now feasible and a concrete possibility [53].

## Conclusions

We are currently advancing in the knowledge of the molecular mechanisms for sex determination in mosquitoes, but many steps are still needed to achieve genetic control of mosquito-borne disease. The biotechnological strategy of producing male-only progeny could be developed for many dipteran species where the SIT is employed. A novel sex separation method obtained through genetic manipulation of mosquitoes is fundamental to improve male-release technological approaches, and it will initiate a more advanced and efficient alternative for mosquito population suppression. Mosquito control strategies based on reducing the number of females or converting them into males may bring many benefits in combating arboviruses that are spreading in tropical and subtropical regions by reducing human-vector contact.

## Abbreviations

CHIKV: Chikungunya virus
CI: Cytoplasmic incompatibility
Dsx: Doublesex
Fru: Fruitless
GSS: Genetic sexing strains
IIT: Incompatible insect technique
IVM: Integrated vector management
RIDL: Release of Insects carrying a Dominant Lethal
SIT: Sterile insect technique
Tra-2: Transformer 2
YF: Yellow fever
ZIKV: Zika virus
